# Modulatory Role of Autophagy in Metformin Therapeutic Activity toward Doxorubicin-Induced Nephrotoxicity

**DOI:** 10.3390/toxics11030273

**Published:** 2023-03-16

**Authors:** Samar A. Antar, Marwa Abd-Elsalam, Walied Abdo, Ahmed Abdeen, Mohamed Abdo, Liana Fericean, Nahed A. Raslan, Samah F. Ibrahim, Asmaa F. Sharif, Amira Elalfy, Hend E. Nasr, Ahmed B. Zaid, Rania Atia, Ahmed M. Atwa, Mohammed A. Gebba, Amany A. Alzokaky

**Affiliations:** 1 Department of Pharmacology and Biochemistry, Faculty of Pharmacy, Horus University-Egypt, New Damietta 34518, Egypt; 2 Center for Vascular and Heart Research, Fralin Biomedical Research Institute, Virginia Tech, Roanoke, VA 24016, USA; 3 Department of Histology, Faculty of Medicine, Kafrelsheikh University, Kafr El-Sheikh 33516, Egypt; 4 Department of Pathology, Faculty of Veterinary Medicine, Kafrelsheikh University, Kafr El-Sheikh 33516, Egypt; 5 Department of Forensic Medicine and Toxicology, Faculty of Veterinary Medicine, Benha University, Toukh 13736, Egypt; 6 Department of Animal Histology and Anatomy, School of Veterinary Medicine, Badr University in Cairo (BUC), Badr City 32897, Egypt; 7 Department of Anatomy and Embryology, Faculty of Veterinary Medicine, University of Sadat City, Sadat City 32897, Egypt; 8 Department of Biology and Plant Protection, Faculty of Agriculture, University of Life Sciences “King Michael I” from Timișoara, Calea Aradului 119, CUI 3487181, 300645 Timisoara, Romania; 9 Clinical Pharmacy Program, College of Health Sciences and Nursing, Al-Rayan Colleges, Medina 42541, Saudi Arabia; 10 Department of Pharmacology and Toxicology, Faculty of Pharmacy (Girls), Al-Azhar University, Cairo 11651, Egypt; 11 Department of Clinical Sciences, College of Medicine, Princess Nourah bint Abdulrahman University, P.O. Box 84428, Riyadh 11671, Saudi Arabia; 12 Department of Forensic Medicine and Clinical Toxicology, Faculty of Medicine, Tanta University, Tanta 31111, Egypt; 13 Department of Histology and Cell Biology, Faculty of Medicine, Benha University, Benha 13518, Egypt; 14 Department of Medical Biochemistry and Molecular Biology, Faculty of Medicine, Benha University, Benha 13518, Egypt; 15 Department of Clinical Pathology, National Liver Institute, Menoufia University, Shibin Elkom 32511, Egypt; 16 Department of Physiology, Faculty of Medicine, Zagazig University, Zagazig 44519, Egypt; 17 Department of Basic Medical Science, Faculty of Applied Medical Science, Al-Baha University, Al-Baha 65779, Saudi Arabia; 18 Department of Pharmacology and Toxicology, Faculty of Pharmacy, Egyptian Russian University, Cairo 11829, Egypt; 19 Department of Anatomy and Embryology, Faculty of Medicine, Benha University, Benha 13518, Egypt

**Keywords:** chemotherapeutic agents, renal injury, NGAL, LC3B, Beclin-1, oxidative damage, inflammatory pathway

## Abstract

Doxorubicin (DOX) is a frequent chemotherapeutic drug used to treat various malignant tumors. One of the key factors that diminish its therapeutic importance is DOX-induced nephrotoxicity. The first-line oral antidiabetic drug is metformin (Met), which also has antioxidant properties. The purpose of our study was to investigate the underlying molecular mechanisms for the potential protective effects of Met on DOX-triggered nephrotoxicity. Four animal groups were assigned as follows; animals received vehicle (control group), 200 mg/kg Met (Met group), DOX 15 mg/kg DOX (DOX group), and a combination of DOX and Met (DOX/Met group). Our results demonstrated that DOX administration caused marked histological alterations of widespread inflammation and tubular degeneration. Notably, the DOX-induced dramatic up-regulation of the nuclear factor-kappa B/P65 (NF-κB/P65), microtubule-associated protein light chain 3B (LC3B), neutrophil gelatinase-associated lipocalin (NGAL), interleukin-1beta (IL-1β), 8-hydroxy-2′ -deoxyguanosine (8-OHdG), and Beclin-1 in renal tissue. A marked increase in the malondialdehyde (MDA) tissue level and a decrease in the total antioxidant capacity (TAC) were also recorded in DOX-exposed animals. Interestingly, Met could minimize all histopathological changes as well as the disruptions caused by DOX in the aforementioned measures. Thus, Met provided a workable method for suppressing the nephrotoxicity that occurred during the DOX regimen via the deactivation of the Beclin-1/LC3B pathway.

## 1. Introduction

Chronic kidney disease (CKD) is a set of chronic progressive nephropathy associated with a steady loss of renal function. Recently, the prevalence of CKD has risen to reach 14%, and it coincided with an increased mortality rate [1]. Unfortunately, cancer is one of the factors leading to the development of CKD either in a direct way or indirectly through the renal toxicity that took place after chemotherapy [2]. Renal injury is a common pathophysiological event associated with cancer patients undergoing chemotherapy [3]. 

Doxorubicin (DOX) is one of the most competent anthracycline drugs against different cancer types. However, its limited efficacy was related to systemic toxicity, particularly kidney injury [4]. It is an anthracycline antibiotic with potent anti-neoplastic function mediated mainly by inhibiting DNA synthesis [5]. It is effective against a broad range of malignancies such as cancers of the lung, breast, ovary, uterus, and uterine cervix in addition to leukemia [6]. The detailed mechanism of DOX-induced nephrotoxicity is still unknown. However, in most known data, DOX altered the hemodynamics within the glomerular tuft, causing the activation of inflammatory signals, prothrombotic angiopathy and crystal nephropathy, and tubular injury [7]. Moreover, the disruption of oxidant-antioxidant homeostasis, free radical overproduction lipid peroxidation, and protein oxidation contribute to DOX-associated nephrotoxicity [8]. The nephrotoxicity of DOX was augmented by a direct breakdown of cell membranes and intercalation with DNA [9].

Autophagy is a lysosomal degradation mechanism for clearing and reusing cytoplasmic components [10]. Autophagy was a physiological process essential for cell survival via combatting cellular stress and scavenging damaged or aged cellular macromolecules [11]. It is well known that intracellular stress including the ascent of reactive oxygen species (ROS), protein misfolding associated endoplasmic reticulum (ER) stress, cellular hypoxia as well as DNA damage, and immunological signaling) urgently stimulate the cascade of the autophagy process. Initiation, nucleation, growth, fusion, and degradation are considered sequential steps in the autophagy process [12]. Several experimental models of kidney malfunction have shown that autophagy is beneficial for pharmacological stimulation and has a protective advantage when autophagy is kept within a certain range [13,14]. Therefore, there is an interplay between autophagy and inflammatory signaling pathway during DOX-induced oxidative stress. It is known that the increased generation of ROS can activate mitogen-activated protein kinases (MAPKs), which induce autophagy by phosphorylation of Bcl-2, with subsequent dissociation from Beclin-1. Hence, Beclin-1 expression may represent the autophagic state of the cell. Moreover, the central role of microtubule-associated protein light chain 3B (LC3B) is in autophagic activity, since it is required for phagophore elongation [15,16].

Metformin (Met), a metabolic regulator, is extensively used with type-II diabetics [17]. Met efficiently maintains metabolic homeostasis via the activation of adenosine 5′-monophosphate-activated protein kinase (AMPK) [18,19]. In addition to its anti-hyperglycemic privilege, it has forceful antioxidant and anti-inflammatory properties [20], anticancer, antiaging, neuroprotective, and cardiovascular protective activities [21]. 

Thus, this study was assigned to assess the possible protective effect of Met against nephrotoxicity inflicted during the DOX regimen and to unveil its underlying antioxidant, anti-inflammatory and anti-autophagy mechanisms involving Beclin-1 and LC3B. 

## 2. Results

### 2.1. Prophylactic Effect of Met on DOX-Induced Changes on Kidney and Body Weights

**Figure 1:**
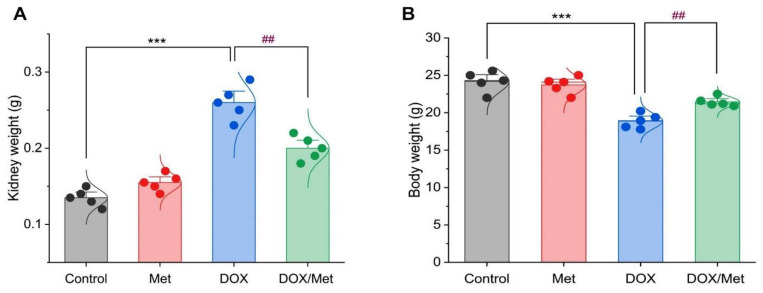
Bar-dot plot of the prophylactic effect of Met on the kidney and body weight changes induced by DOX-therapy. Prophylactic treatment with Met for 3 weeks decreased both kidney weight (**A**) and body weight (**B**) in comparison with the DOX group. Student’s *t*-test was used for statistical comparisons. Data are expressed as mean ± SEM. Colored dots indicate each individual value and colored curves represent the distribution of each data point. *** *p* < 0.001 vs. control group and ^##^ *p* < 0.01 vs. DOX group.

By weighing the kidneys of the normal and Met groups, it was noted that there was no discernible difference. Furthermore, DOX therapy resulted in a noticeable increase in kidney weight by 48.07% and 40.38% compared to normal or Met groups, respectively (*p* < 0.001). Meanwhile, a significant decrease in weight was observed in the Met prophylactic group when compared to the DOX group (*p* < 0.001). The co-therapy group significantly reduced kidney weight by 23.07% (Figure 1A).

Regarding animal body weight, there was no significant difference between the control group and the Met group. While the weight of animals within the DOX group was greatly elevated by 28.07% and 25.06% when compared to normal and Met groups, respectively. Furthermore, compared to the DOX group, the co-administration of DOX and Met demonstrated a substantial decrease in animal weight of 13.66% (Figure 1B).

### 2.2. Prophylactic Effect of Met on DOX-Induced Changes on Serum Creatinine and Urea Levels

**Figure 2:**
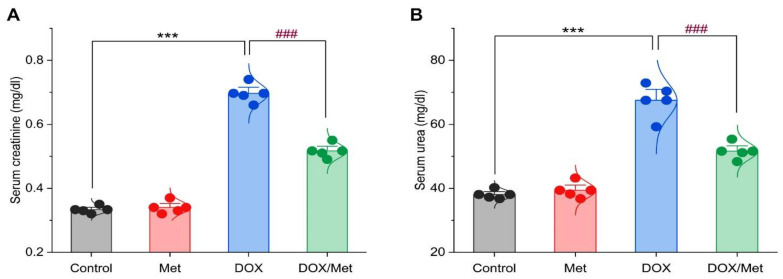
Bar-dot plot of the prophylactic effect of Met on DOX-induced renal dysfunction. Prophylactic treatment with Met for 3 weeks decreased serum creatinine (**A**) and urea (**B**) in the DOX-nephrotoxic mice. Student’s *t*-test was used for statistical comparisons. Data are expressed as mean ± SEM. Colored dots indicate each individual value and colored curves represent the distribution of each data point. *** *p* < 0.001 vs. control group and ^###^ *p* < 0.001 vs. DOX group.

As depicted in Figure 2, DOX induced a renal injury which was indicated by a remarkable increase in serum creatinine and urea levels. The comparison between the diseased group and control and Met groups showed a marked elevation of the creatinine concentration by 103.65% and 105.14%, respectively. In the same line, the urea recorded high levels at rates of 73.20% and 68.88% when compared with the control and Met groups, respectively. However, the combination between DOX and Met showed significant reductions in creatinine and urea levels by 28.31% and 24.62%, respectively, in comparison with the DOX group.

### 2.3. Prophylactic Effect of Met on DOX-Induced Changes on Histopathological Picture

**Figure 3:**
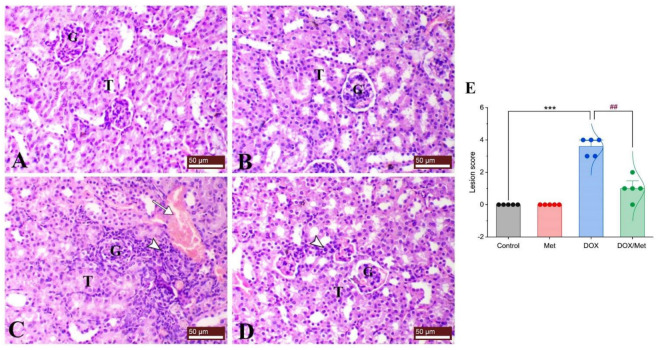
Protective effect of Met against histological alterations in renal tissue injured by DOX. (**A**) control group, (**B**) Met-treated group, (**C**) DOX-intoxicated group, and (**D**) DOX/Met group. (**E**) Bar-dot plot of the histopathological scoring. Tailed arrows indicate vascular congestion and arrowhead indicates interstitial inflammatory cell infiltrations. The Mann–Whitney U test was used for statistical comparisons. Data are expressed as mean ± SEM. Colored dots indicate each individual value and colored curves represent the distribution of each data point. *** *p* < 0.001 vs. control group and ^##^ *p* < 0.01 vs. DOX group. (H and E stained; G, glomerulus; T, renal tubule; bars = 50 µm).

Regarding the histological pictures of renal tissue of both control and Met groups, the renal cortex showed normal glomeruli and renal tubules. The renal tubules of different sections either proximal or distal demonstrated normal renal tubular epithelial lining. The kidney of the DOX group exhibited features of tubulointerstitial nephritis associated with nephrotic changes of the renal tissues as degeneration of renal tubular epithelium and interstitial infiltration of mononuclear cells consisting of lymphocytes and macrophages. On the other hand, the DOX/Met group revealed a marked decrease in the degenerative and inflammatory changes within the renal tissues (Figure 3).

### 2.4. Protective Effect of Met on DOX-Induced Changes on Renal Neutrophil Gelatinase-Associated Lipocalin (NGAL) Content

**Figure 4:**
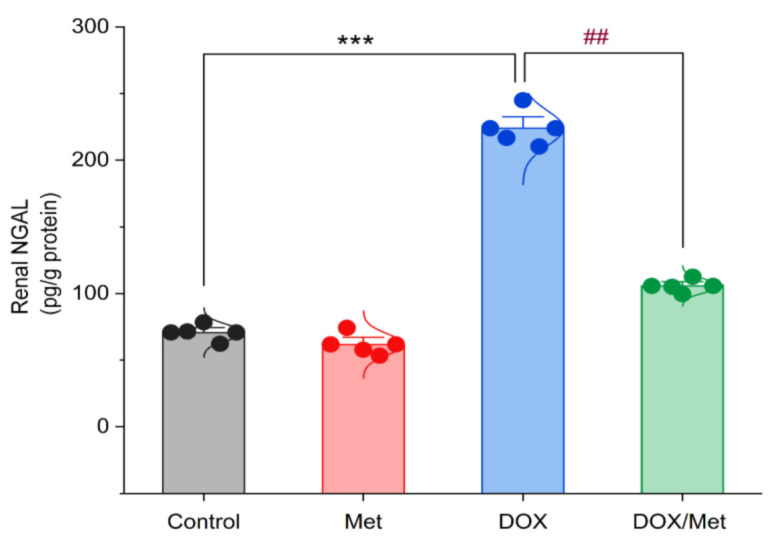
Bar-dot plot of the prophylactic effect of Met on DOX-induced changes on renal NGAL content. Student’s *t*-test was used for statistical comparisons. Data are expressed as mean ± SEM. Colored dots indicate each individual value and colored curves represent the distribution of each data point. *** *p* < 0.001 vs. control group and ^##^ *p* < 0.01 vs. DOX group.

In Figure 4, the renal NGAL content was nearly similar in the control and Met groups. Following DOX administration, a significant increase in the renal NGAL content by 68.42% compared with the control group (*p <* 0.001). The combination of DOX and Met markedly reduced the renal NGAL content by 52.8% in comparison with the DOX group (*p* < 0.001).

### 2.5. Prophylactic Effect of Met on DOX-Induced Changes on Renal Malondialdehyde (MDA), Total Antioxidant Capacity (TAC), and 8-hydroxy-2′-deoxyguanosine (8-OHdG) Contents

**Figure 5:**
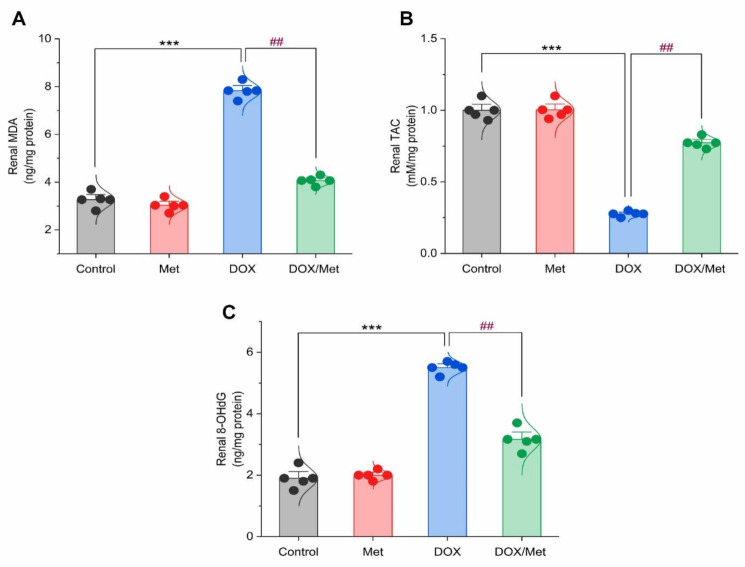
Bar-dot plot of the prophylactic effect of Met on DOX-induced oxidative stress in renal tissue. Prophylactic treatment with Met for 3 weeks decreased renal MDA (**A**) along with increased renal TAC (**B**) and renal 8-OHdG (**C**) content in DOX-induced nephrotoxic mice. Student’s *t*-test was used for statistical comparisons. Data are expressed as mean ± SEM. Colored dots indicate each individual value and colored curves represent the distribution of each data point. *** *p* < 0.001 vs. control group and ^##^ *p* < 0.01 vs. DOX group.

The DOX group showed a marked increase in the MDA renal content by 58.38% in comparison with the control group. While diseased animals treated with Met resulted in a significant decrease in MDA renal content by 48.07% compared to the DOX group (Figure 5A). Following the administration of DOX, renal TAC content significantly decreased by 261% when compared with the control group. Likewise, Met prophylactic resulted in a significant increase in renal TAC when compared to the DOX group. Met treatment with a dose of 200 mg/kg caused a significant rise in renal TAC content by 179.47%. Unfortunately, this value was still significantly lower than the control group (Figure 5B). 

The 8-OHdG content was markedly increased in the DOX group by 65.45% compared to the normal group (*p <* 0.001). The Met prophylactic effect reduced renal 8-OHdG content. The prophylactic group displayed a significant reduction in renal 8-OHdG by 42.41% compared to the DOX group (Figure 5C). 

### 2.6. Prophylactic Effect of Met on DOX-Induced Inflammation

**Figure 6:**
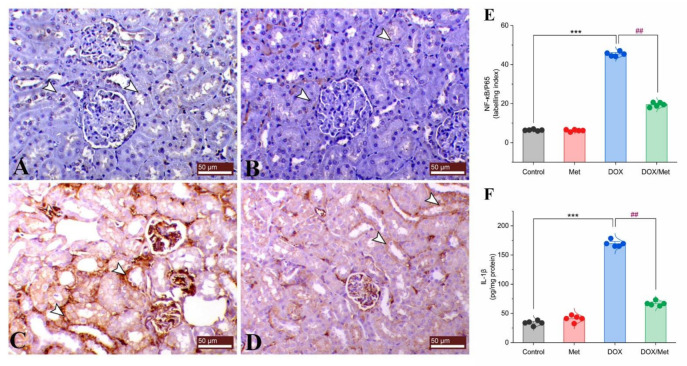
Prophylactic effect of Met on DOX-induced inflammation. The figure shows the protein expression of NF-κB/P65 by immunostaining in control (**A**), Met (**B**), DOX (**C**), and DOX/Met (**D**) groups (arrows indicate the positive stained area; bars = 50 µm). (**E**) Bar-dot plot of the semi-quantitative analysis of NF-κB/P65 expression levels in renal tissue obtained by immunostaining. (**F**) Bar-dot plot of the renal concentration of IL-1β after Met and/or DOX treatments. Student’s *t*-test was used for statistical comparisons. Data are expressed as mean ± SEM. Colored dots indicate each individual value and colored curves represent the distribution of each data point. *** *p* < 0.001 vs. control group and ^##^ *p* < 0.01 vs. DOX group.

Control and sham groups showed slight immune expression of nuclear factor-kappa B/P65 (NF-κB/P65) within the renal tubular lining epithelium (Figure 6A and Figure 6B, respectively). While the DOX group showed marked both cytoplasmic and nuclear immunostaining of NF-κB/P65 within the renal tubular epithelium (Figure 6C). The kidney of the prophylactic group showed marked downregulation of renal NF-κB/P65 expression (Figure 6D,E).

Interleukin (IL)-1β is known as a signaling proinflammatory biomarker. In comparison with the normal group, DOX treatment caused a considerable rise in renal IL-1β level of 80.11%. On the other hand, Met treatment caused a significant reduction in renal IL-1β concentration. When compared to the DOX group, prophylactic Met (200 mg/kg, orally) administration showed a substantial decrease in renal IL-1β level by 60.54% (Figure 6F).

### 2.7. Prophylactic Effect of Met on Renal LC3B and Beclin-1 Expression

**Figure 7:**
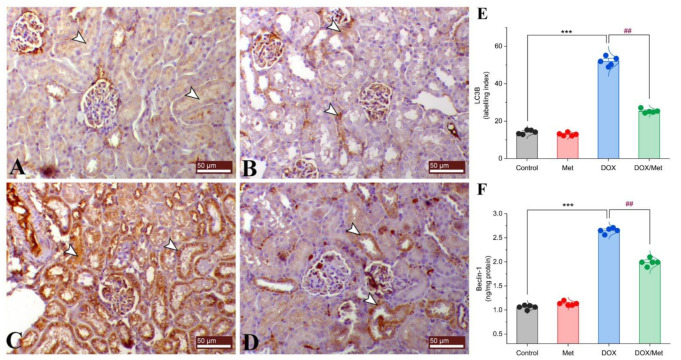
Prophylactic effect of Met on renal LC3B and Beclin-1 expression levels. The figure shows the protein expression of LC3B by immunostaining in control (**A**), Met (**B**), DOX (**C**), and DOX/Met (**D**) groups (arrows indicate the positive stained area; bars = 50 µm). (**E**) Bar-dot plot of the semi-quantitative analysis of LC3B expression levels in renal tissue obtained by immunostaining. (**F**) Bar-dot plot of the renal concentration of Beclin-1 after Met and/or DOX treatments. Student’s *t*-test was used for statistical comparisons. Data are expressed as mean ± SEM. Colored dots indicate each individual value and colored curves represent the distribution of each data point. *** *p* < 0.001 vs. control group and ^##^ *p* < 0.01 vs. DOX group.

The control and Met groups exhibited negative mild cytoplasmic immunostaining for renal LC3B (Figure 7A and Figure 7B, respectively). On the contrary, DOX group showed a dramatic increase in the expression level of LC3B compared to controls (Figure 7C). However, combined therapy of DOX and Met could noticeably decrease the LC3B expression (Figure 7D,E). 

Beclin-1 protein expression was significantly elevated by approximately 60%, 57.24% in the DOX-treated mice compared with the control and Met groups. Prophylactic treatment with Met reduced the renal Beclin-1 protein expression by 24.79%, compared with the DOX group (Figure 7F).

### 2.8. Hierarchical Clustering Heatmap and Variable Important Project (VIP) Score

**Figure 8:**
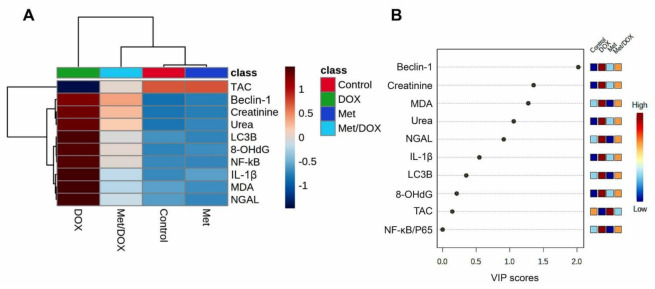
Hierarchical clustering heatmap and variable important project (VIP) scores provide an intuitive visualization of all data sets. (**A**) Clustering heatmap for the variable averages and different treatments; The concentration levels are represented by each colored cell on the map, with varied averages in rows and various treatments in columns. (**B**) Variable important project (VIP) score; the extent to which different factors contribute. A colored scale with the highest (red color) and lowest (blue color) value designates the contribution strength (blue color). Dark red (highest value) is at the top of the gradient scale, while blue is at the bottom (lowest value).

The clustering heatmap depicted in Figure 8A provides an intuitive visualization of all data sets which summarizes the concentration values of all measured biochemical parameters in response to different treatments. The gradient scale of the heatmap showed the highest red-intensity-colored cells that indicating elevations in the concentration levels of creatinine, urea, MDA, 8-OHG, NF-κB/P65, IL-1β, Beclin-1, and LC3B along with dark blue cell corresponding to the lowest level of TAC in the DOX-treated animals compared to the controls and Met-treated animals. Meanwhile, the cell color intensity of the DOX/Met group showed intermediate color intensity at all measured variables suggesting the occurrence of improvements after Met therapy in DOX-injured mice.

Next, to determine the most influencing parameter in response to different treatments in the current study, VIP score was conducted. As depicted in Figure 8B, Beclin-1, creatinine, MDA, urea, NGAL, and IL-1β were the most important variables influencing the DOX-induced renal injury.

## 3. Discussion

DOX is one of the most potent anthracyclines for treating various cancers. However, the DOX regimen is frequently linked to nephrotoxicity [4]. Despite the potential use of Met as an anti-hyperglycemic medication used to treat type-II diabetes, it has been reported to have antioxidant and anti-inflammatory therapeutic activities [20]. Herein, the prophylactic effect of Met against DOX-induced kidney damage was elucidated.

Expectedly, DOX could exert renal damage indicated by elevated levels of serum creatinine and urea. A dramatic increase in the NGAL was also noticed in DOX-treated animals. It is well known that NGAL excretion in the urine is a recognized indicator for tubular epithelial injury. Along with these, the current histopathological examination exhibited the existence of tubular degeneration and loss of tubular epithelium which was the reason that contributed to the elevated creatinine, urea, and NGAL levels.

It has been hypothesized that the primary process driving the nephrotoxic impact of DOX is oxidative stress and its downstream events such as free radical overproduction, lipid peroxidation, and antioxidant enzymes depletion [22,23]. Oxidative damage is known to develop under the condition where there is an imbalance between the generated reactive oxygen species (ROS) and the cellular antioxidant competence [24]. The release of ROS (mainly; superoxide anion radical (O_2_^•−^), hydrogen peroxide (H_2_O_2_), hydroxyl radical (OH^•^), and nitric oxide (NO) into the cytosol can cause further damage to other cellular macromolecules including disintegration of lipid bilayer membranes, mitochondrial dysfunction, protein misfolding, endoplasmic reticulum (ER) stress, and DNA damage [23]. The problem is made worse by the fact that OH^•^ can distantly target the cellular molecules, specifically the lipid content of cell membranes, leading to lipid peroxidation and the subsequent generation of another damaging compound (particularly, MDA). Consistently, our data revealed a marked increase in MDA levels along with depletion in the TAC; thereby, the cell becomes more vulnerable to the damaging effect of ROS and MDA. Therefore, the noticed tubular degeneration and loss of tubular epithelium might be attributed to the generalized damage brought on by the buildup of the damaging radical (ROS and MDA). Moreover, oxidative species have been implicated in disrupting the nephron’s excretory function, which leads to an imbalance in homeostasis and the accumulation of metabolic waste products. It is widely known that renal cells include a large number of mitochondria, providing the adenosine triphosphate (ATP) needed for active transport and the removal of metabolic byproducts. In the meantime, those harmful radicals inhibit the mitochondrial oxidative phosphorylation required for ATP synthesis. We therefore strongly suspect that the observed renal impairment, as indicated by increased levels of creatinine, urea, and NGAL, was caused by oxidative damage brought on by DOX.

In the current investigation, DOX administration substantially enhanced the NF-κB/P65 and IL-1β expression levels in the renal tissues which is consistent with the previously published data [25]. It is interesting to note that inflammation is the main contributor to DOX-induced toxicity and that it is proposed to activate a product of multiple regulatory mechanisms working in concert [26]. These sequences are listed briefly as the following; by the breakdown of the inhibitor of kappa-B (IkB-α), DOX promoted the phosphorylation and translocation of NF-κB/P65 to the nucleus triggering the transcription of the pro-inflammatory cytokines [27]. A growing body of evidence suggests that upon activation of NF-κB/P65, the pro-inflammatory response was associated with ROS which leads to endothelial dysfunction; thereby, a dozen amounts of cytokines and chemokines such as IL-1β, tumor necrosis factor-alpha (TNF-α), and monocyte chemoattractant protein-1 (MCP-1) are activated, which adds to the escalation of inflammation [28]. Furthermore, an extended inflammatory response, tissue damage, and transcription of numerous genes implicated in inflammatory and apoptotic responses are significantly influenced by NF-κB/P65 [29]. There is a great assumption that ROS are positively correlated with the initiation of the inflammatory response via activation of the NF-κB/P65 and mitogen-activated protein kinase (MAPK) pathways [30]. Thus, our data strongly proposed that this inflammatory reaction was a downstream event to the DOX-induced oxidative stress.

It is well known that the autophagy process, including the removal of protein aggregates and damaged organelles, improving cellular energy production, and reduction the endoplasmic reticulum stress, is required for maintaining cell physiology and survival. Thus, for the preservation of kidney function, basal autophagy appears to be crucial for the survival of podocytes, proximal tubular epithelial cells, glomerular mesangial cells, and glomerular endothelial cells [31]. As previously reported, the autophagy mechanisms are closely related to the inflammatory signaling pathways. The inhibitor of B protein maintains NF-κB in an inactive state in the cytoplasm, where it has a vital regulatory role in the inflammatory response. IκB kinase (IKK) joins forces with TGF-activated kinase 1 (TAK1), MAP3K7-binding protein 2 and 3, and TAK1 to form a complex that inhibits IκB degradation [32]. Notably, TGF-activated the kinase 1-binding protein 2/3, which considers one of the TAK1 cofactors, which bind to Beclin-1 and promote autophagy. It is worth noting that an equilibrium between the IKK-NF-κB pathway and the autophagy process [33]. Additionally, it is supposed that DOX interferes with renal autophagy in autophagy-deficient situations by suppressing the biogenesis and function of the lysosomes through aberrant transcription factor-EB (TFEB). By the earlier discovery, the disruption of cardiac autophagic mechanisms results in ROS overproduction and dissociation, which promote mitochondria-mediated apoptosis and death [34,35]. It has been shown that increased mitochondrial ROS production causes oxidative damage to skeletal muscle proteins, which increases autophagy by disrupting ER homeostasis and induction of the unfolded protein response [36].

Furthermore, Beclin-1 is also a critical determinant of whether cells act in autophagy [37]. Beclin-1 binds together with other cofactors to form the BCL2-interacting coiled-coil protein (BECN1)-phosphatidylinositol 3-kinase catalytic subunit type 3 (PIK3C3)-phosphoinositide-3-kinase regulatory subunit 4 (PIK3R4) complex, which promotes the development of autophagosomes [38]. LC3 attaches to autophagosomes, therefore, the LC3-II/I ratio rises during autophagy development, followed by a progressive conversion of LC3-I into LC3-II. Thus, LC3 is considered a potential marker for the detection of autophagy flux [39]. In the current study, there was a significant up-regulation in Beclin-1 content and LC3B expression associated with DOX administration. Our findings were in the same line with the former reports which revealed initiation of autophagy and the formation of autophagosomes in cardiac cells in response to DOX insult [40].

Hence, Met has antioxidant and anti-inflammatory activities, and it could significantly restore kidney function in DOX-treated animals shown in the measured creatinine, urea, and NGAL. Met has also an additive advantage through reduction of the degree of insulin resistance, improvement of the renal proximal tubular glomerular filtration function, and lowering the blood glucose [41]. Met treatment was associated with a decreased inflammatory state in diabetics through the reduction of serum NGAL levels in diabetic patients [42]. The present data revealed that the Met therapy dramatically lowered the pro-inflammatory mediators including NF-κB and IL-1β, which confirmed the anti-inflammatory properties [43]. It has been documented that hyperglycemia is accompanied by the activation of an inflammatory cascade including up-regulation of NF-κB and elevated MCP-1 expression levels [44]. Interestingly, the anti-hyperglycemic property of Met would be another possible factor in mitigating the DOX-induced inflammation in the current study [44]. Kim et al. have demonstrated the anti-inflammatory competence of Met during chronic kidney disease via reduction of the IL-1β and transforming growth factor- beta (TGF-β1) levels [45].

Remarkably, Met protects young C57BL/6 mice fed a high-fat diet from obesity-induced kidney damage [46]. The underlying mechanism involves the activation of renal AMPK and fatty acid oxidation. It significantly reduces macrophage infiltration and glomerular mesangial matrix growth in mice kidney tissues. In C57BL/6 mice with folic acid-induced nephropathy, Met guards against renal inflammatory responses and tubulointerstitial fibrosis [47]. Moreover, when the kidney of Sprague-Dawley rats was subjected to ischemia and renal arteriovenous perfusion and preconditioned with Met, it showed a marked reduction in the renal tubular epithelial cell necrosis and inflammation [48]. These data supported our histopathological findings that revealed a marked decrease in the tubulointerstitial inflammation in the diseased group treated with Met. The protective effect of the Met could be exerted also by a restoration of antioxidant enzymes through an increase of superoxide dismutase (SOD) activity and glutathione peroxidase (GSH) levels and mitigation of inflammation through inhibition of the inflammatory mediators [49,50].

In addition, DOX-exposed animals treated with Met showed a significant decrease in the levels of Beclin-1 and LC3B. These findings are consistent with the role of Met in the modulation of different signaling pathways, including the adenosine monophosphate-activated protein kinase (AMPK) pathway, the Hedgehog pathway, and microRNAs and autophagy block through miR-570-3p, miR-142-3p, and miR-3127-5p [51]. The AMPK/NF-κB pathway was one of the AMPK-related pathways that was associated with the inhibition of the autophagy mechanisms [52]. The activation of NF-κB can directly promote the process of autophagy. Therefore, the suppression of NF-κB activity could efficiently prevent autophagy through activation of AMPK. A recent study found that Met administration causes significant inhibition of NF-κB/P65 nuclear translocation in pulmonary arterial hypertension [53], which suggests the role of autophagy-suppressing mechanisms in the inhibition of AMPK/NF-κB signaling pathway. Consistently, our study documented the potential use of Met in modulating the fate of autophagy in the DOX-nephrotoxic mouse model. 

**Figure 9:**
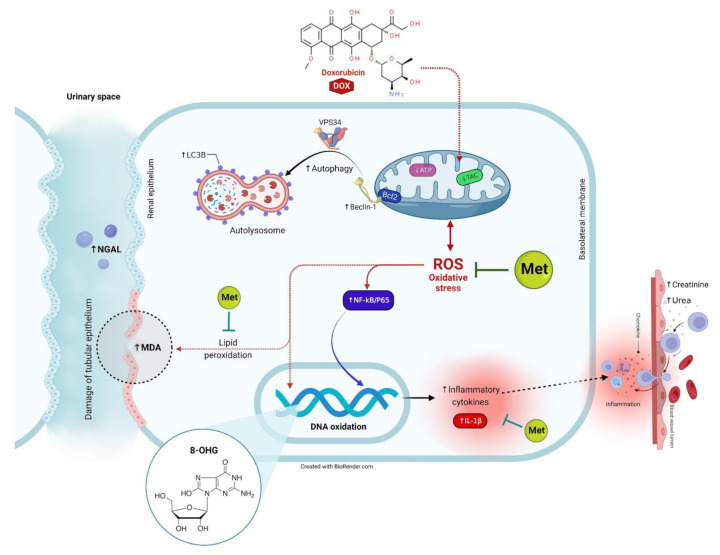
The proposed mechanisms located behind the protective effect of Met against DOX-induced renal injury.

The clustering heatmap provides an intuitive visualization of the concentration values that could potentially discriminate the DOX-treated group from other treatments. Wherein, the DOX showed the occurrence of severe alterations was confirmed by the great differences shown in the concentration values of all variables when compared to the control and Met-treated animals. However, the supplementation of Met to the DOX-injured animals exhibited markable improvements exhibited by the intermediate intensity of the concentration value of all variables as depicted in Figure 8. The VIP score affirms the influencing impact of the selected parameters in the current study which in turn strengthens our hypothesis that the autophagy indicators (mainly, Beclin-1) followed by inflammatory and oxidative stress markers were the main modulator in the Met ameliorating activity toward the DOX-induced nephrotoxicity. Additionally, the information gleaned from the VIP score may serve as possible markers for keeping track of patients receiving the DOX treatment, since the dose of Met in humans ranged from 500 to 2550 mg according to the age, health, hepatic and the renal complications, and the type of diabetes. It is encouraged to conduct a further experiment to investigate the Met at different doses for providing more details about Met dose-response that might simulate those used in humans. The proposed mechanisms located behind the protective effect of Met against Dox-induced renal injury are summarized in Figure 9.

## 4. Materials and Methods

### 4.1. Drugs

DOX (Adriamycin^®^ vial, each ml contains 2 mg) was purchased from EIMC United Pharmaceuticals Company (Cairo, Egypt). Met powder was purchased from Sigma-Aldrich (St. Louis, MO, USA). 

### 4.2. Animal Study

In the current experiment, adult male albino mice (20–25 g) were obtained from the laboratory animal house of the Modern Veterinary Office Faculty of Veterinary Medicine, Cairo University, Cairo, Egypt. Animals were kept in well-acclimatized environmental conditions of a 12 h light and dark cyclic conditions and at a temperature of 25 ± 1 °C. Throughout the study, both food and water were accessed ad-libitum. The research protocol was following the Canadian Council on Animal Care Guidelines and was approved by the committee of Research Ethics, Faculty of Veterinary Medicine, Kafrelsheikh University (KFS/12/21).

**Figure 10:**
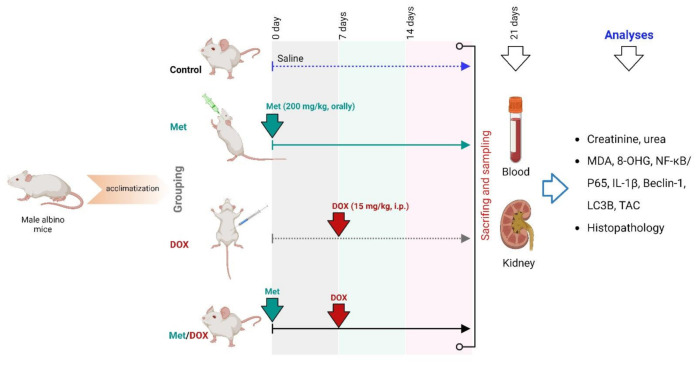
Experimental design.

Fourteen days later, all animals were randomly categorized into four groups (five mice per each group). The first group acted as a control group; received only saline. The second group received a daily dose of Met (200 mg/kg, orally) [54]. A single dose of DOX (15 mg/kg, i.p.) was given to the third group to induce a renal injury [55]. This DOX dose was corresponding to the toxic cumulative dose in human which is 550 mg/m^2^. The fourth group was administrated a combination of DOX and Met. Treatment with Met was started one week ahead of the DOX administration and continued for 14 days as described in Figure 10.

Twenty-four h after the last drug administration, all animals were euthanized under deep anesthesia via thiopental sodium at a dose of 50 mg/kg. A blood sample was obtained from the abdominal aorta, allowed to be clotted for 10 min and then sera were separated after centrifugation for 10 min at 3000 rpm. After that, animals were dissected, kidneys were harvested and then one kidney was fixed in 10% paraformaldehyde solution for histopathological and immunohistochemical investigation. The remaining kidney was frozen at −80 °C for ELISA assays.

### 4.3. Assessment of Kidney Function Tests

According to the commercially available kits, serum creatinine and urea were assessed in accordance with their manufacturer’s instructions (ab204537 and ab83362, respectively; Abcam, Cambridge, UK).

### 4.4. Assessment of NGAL

Assay of NGAL was performed using the manufacturer’s instructions provided by the commercial available ELISA kit (Mouse NGAL, cat No. E-EL-M0828, Elabscience, Houston, TX, USA).

### 4.5. Assessment of Oxidant/Antioxidant Status Biomarkers in Tissue

The oxidative stress parameters including MDA (Mouse MDA, cat No. LS-F28474, LSBio, WA, USA), TAC (Mouse TAC, cat No. K025, FineTest, Wuhan, China), and 8-OHdG (Mouse 8-OHdG, cat No. EM 1636, AMSBIO LLC, Cambridge, MA, USA) contents were measured in the renal homogenate using the available commercial ELISA assay kits. Test procedures were carried out according to the provided manufacturer’s instructions.

### 4.6. Assessment of Beclin-1 and IL-1β Contents in Tissue

Beclin-1was determined using the commercially available ELISA assay kits (Mouse Beclin-1, cat No. LS-F10930, LSBio Co., Seattle, WA, USA). The renal tissue content of IL-1β was assessed using a Mouse IL-1β ELISA kit (cat No. SEA563Mu, Cloud-Clone Crop Co., Wuhan, China).

### 4.7. Histopathology and Immunohistochemistry of NF-κB/P65 and LC3B Protein

The fixed tissue samples were rinsed down in tap water for 10 min, dehydrated in ascending grades of ethyl alcohol, and cleared in xylene. After that, they were immersed in molten paraffin at 58–62 °C. About 5 µm histological sections were made and stained with hematoxylin and eosin. The slides were ready to examine under a bright-field microscope. 

The immunohistochemical staining technique was performed by Khalil et al. [56]. Sections were dewaxed and set in a 0.05 M citrate buffer, pH 6.8 solution for antigen retrieval. Next, these sections were placed in a 0.3% H_2_O_2_ solution for the deactivation of endogenous peroxides followed by protein blocking. Then, sections were incubated with an anti-LC3B antibody, an autophagosome marker (cat No. ab48394, Abcam, CA, USA), and anti-NF-ĸB/P65 (cat No. sc-8008, Santa Cruz, CA, USA) at a dilution rate of 1:100. The slides were then incubated with secondary goat anti-rabbit antibody (cat No. K4003, EnVision+TM System HRP polymer; Dako, Japan) for polyclonal antibodies for 30 min at room temperature following phosphate buffered saline rinse. Slides were then covered with a drop of DAB kit solution and eventually counterstained with Mayer’s hematoxylin. The staining labeling indices of the antibodies were presented as the mean of the percent of positive area/mm^2^.

### 4.8. Statistical Analyses

The numerical data were expressed as mean ± SEM. Statistical analysis was conducted using Student’s *t*-test and Mann–Whitney U test for two-group statistical comparison of parametric and nonparametric data, respectively. *p* < 0.001 was accepted as being statistically significant. Data were plotted by the OriginPro software (version 2019b). RStudio (R version 4.0.2) was used to create the clustering heatmap and the variable importance in projection (VIP) score. The “pheatmap, dendextend, gplots, colorspace, vip” packages were used to generate those analyses.

## 5. Conclusions

The present research demonstrated the possible renal protection against DOX-inflicted injury through oral Met treatment. Marked improvements in kidney function by Met administration via reduction of oxidative stress, suppression of pro-inflammatory cytokines, and reduction of DOX-induced autophagy were documented. The therapeutic potential of Met was possibly attributed mainly to its antioxidant and anti-inflammatory properties.

## Data Availability

The corresponding authors can provide the data used to verify the findings of this research upon request.
